# A double blind, randomized, placebo controlled study of the efficacy and safety of 5-Loxin^® ^for treatment of osteoarthritis of the knee

**DOI:** 10.1186/ar2461

**Published:** 2008-07-30

**Authors:** Krishanu Sengupta, Krishnaraju V Alluri, Andey Rama Satish, Simanchala Mishra, Trimurtulu Golakoti, Kadainti VS Sarma, Dipak Dey, Siba P Raychaudhuri

**Affiliations:** 1 Cellular and Molecular Biology Division, Laila Impex R&D Center, Jawahar Autonagar, Vijayawada, 520 007 India; 2 Pharmacology Division, Laila Impex R&D Center, Jawahar Autonagar, Vijayawada, 520 007 India; 3 Department of Orthopedics, Alluri Sitarama Raju Academy of Medical Sciences (ASRAM), National Highway 5, Eluru, 534 002 India; 4 Department of Internal Medicine, Alluri Sitarama Raju Academy of Medical Sciences (ASRAM), National High way 5, Eluru, 534 002 India; 5 Drug Discovery and Development Division, Laila Impex R&D Center, Jawahar Autonagar, Vijayawada, 520 007 India; 6 Department of Statistics, Prakasam Road, SV University, Tirupati, 517 592 India; 7 Department of Statistics, 215 Glenbrook Road, University of Connecticut, Storrs, Connecticut 06269, USA; 8 Department of Medicine, Division of Rheumatology, Allergy and Immunology, School of Medicine, U C Davis and VA Medical Center Sacramento, Hospital Way, Mather, California 95655, USA

## Abstract

**Introduction:**

5-Loxin^® ^is a novel *Boswellia serrata *extract enriched with 30% 3-O-acetyl-11-keto-beta-boswellic acid (AKBA), which exhibits potential anti-inflammatory properties by inhibiting the 5-lipoxygenase enzyme. A 90-day, double-blind, randomized, placebo-controlled study was conducted to evaluate the efficacy and safety of 5-Loxin^® ^in the treatment of osteoarthritis (OA) of the knee.

**Methods:**

Seventy-five OA patients were included in the study. The patients received either 100 mg (n = 25) or 250 mg (n = 25) of 5-Loxin^® ^daily or a placebo (n = 25) for 90 days. Each patient was evaluated for pain and physical functions by using the standard tools (visual analog scale, Lequesne's Functional Index, and Western Ontario and McMaster Universities Osteoarthritis Index) at the baseline (day 0), and at days 7, 30, 60 and 90. Additionally, the cartilage degrading enzyme matrix metalloproteinase-3 was also evaluated in synovial fluid from OA patients. Measurement of a battery of biochemical parameters in serum and haematological parameters, and urine analysis were performed to evaluate the safety of 5-Loxin^® ^in OA patients.

**Results:**

Seventy patients completed the study. At the end of the study, both doses of 5-Loxin^® ^conferred clinically and statistically significant improvements in pain scores and physical function scores in OA patients. Interestingly, significant improvements in pain score and functional ability were recorded in the treatment group supplemented with 250 mg 5-Loxin^® ^as early as 7 days after the start of treatment. Corroborating the improvements in pain scores in treatment groups, we also noted significant reduction in synovial fluid matrix metalloproteinase-3. In comparison with placebo, the safety parameters were almost unchanged in the treatment groups.

**Conclusion:**

5-Loxin^® ^reduces pain and improves physical functioning significantly in OA patients; and it is safe for human consumption. 5-Loxin^® ^may exert its beneficial effects by controlling inflammatory responses through reducing proinflammatory modulators, and it may improve joint health by reducing the enzymatic degradation of cartilage in OA patients.

**Trail Registration:**

(Clinical trial registration number: ISRCTN05212803.)

## Introduction

Osteoarthritis (OA) is the commonest form of inflammatory joint disease, characterized by articular cartilage degradation with an accompanying peri-articular bone response [1,2]. OA affects nearly 21 million people in the USA, accounting for 25% of visits to primary care physicians. It is estimated that 80% of the population will have radiographic evidence of OA by age 65 years, although only 60% of those will be symptomatic [3]. Clinical manifestations of OA of the knee include pain in and around the joint, stiffness of the joint after rest, crepitating on motion and limited joint motion, among others [4]. Current recommendations for managing OA focus on relieving pain and stiffness and improving physical function as important goals of therapy [5,6]. Currently available medication regimens for most cases include nonopioid analgesics such as acetaminophen and nonsteroidal anti-inflammatory drugs (NSAIDs), including cyclo-oxygenase II inhibitors. These pharmaceutical agents can reduce both pain and inflammation quite effectively, but long-term use of NSAIDs has been found to be associated with enhanced risk for gastrointestinal bleeding, hypertension, congestive heart failure and renal insufficiency, among other adverse effects [7-9]. Because of the high incidence of adverse events associated with both nonselective and cyclo-oxygenase II selective NSAID therapy, effective and safer alternative treatments for OA are urgently needed.

In recent years, the gum resin extracted from the ancient herb *Boswellia serrata *has gained much attention as a potent anti-inflammatory, anti-arthritic and analgesic agent [10,11]. 3-O-acetyl-11-keto-beta-boswellic acid (AKBA) is the most active component of *Boswellia *extract and has been demonstrated to be a potent inhibitor of 5-lipoxygenase (5-LOX), which is a key enzyme in the biosynthesis of leukotrienes from arachidonic acid in the cellular inflammatory cascade [12,13].

5-Loxin^® ^is a novel *B. serrata *extract enriched to 30% AKBA (US Patent publication no.: 2004/0073060A1). In the carrageenan-induced inflammation model, 5-Loxin^® ^treatment yielded significant improvement in paw inflammation in albino Wister rats [14]. Cell based *in vitro *studies and *in vivo *experiments conducted in Sprague-Dawley rats suggest that 5-Loxin^® ^can inhibit proinflammatory cytokines such as tumour necrosis factor-α, interleukin-1β (unpublished data, Sengupta K, Alluri KV, and Golakoti T). Furthermore, Affimatrix gene chip analysis demonstrates 5-Loxin^® ^can potentially inhibit the tumour necrosis factor-α induced gene expression of matrix metalloproteinases (MMPs), adhesion molecules such as intercellular adhesion molecule-1, vascular cell adhesion molecule-1, and mediators of apoptosis in human microvascular endothelial cells [14]. Importantly, extensive acute and dose-dependent subchronic safety experiments on rats demonstrate that 5-Loxin^® ^does not exhibit toxic manifestations, even at a dose 2,000 to 3,000 times higher than the human equivalence dose [15]. In addition, 5-Loxin^® ^does not exhibit genotoxicity in the standard AMES bacterial reverse mutation assay (INTOX, 375, Urawade, Pirangut-Urawade Road, Tal. Mulshi, Pune – 412108, India; study no. 4477/05).

Although a significant number of clinical study reports support the anti-inflammatory and anti-arthritic properties of *Boswellia *extract [16-19], to the best of our knowledge no reports on the efficacy of AKBA-enriched 5-Loxin^® ^in OA in humans have been published. Therefore, in the present double-blind and placebo-controlled clinical study, we sought to evaluate the efficacy and safety of 5-Loxin^® ^in treatment of OA of the knee. We assessed the effectiveness of 100 mg/day and 250 mg/day 5-Loxin^® ^on pain, joint stiffness and mobility in OA patients. We also explored the effect of 5-Loxin^® ^on the cartilage degrading enzyme MMP-3 in OA patients treated with 5-Loxin^®^.

## Materials and methods

### Recruitment of patients

**Figure 1:**
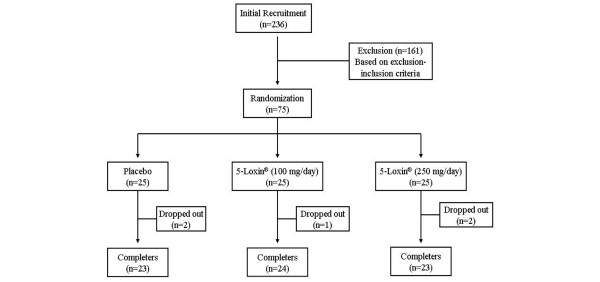
Flow chart of the patients who participated in the clinical trial. Evaluations of physical activity and pain scores, serum biochemistry, haematology, urine biochemistry and proinflammatory cytokines were done at baseline (day 0) and on days 7, 30, 60 and 90 during follow up. Assessments of matrix metalloproteinase-3 were done on days 0 and 90 only.

This trial was performed at Alluri Sitarama Raju Academy of Medical Sciences (ASRAM), Eluru, Andhra Pradesh, India from July 2006 to October 2006 (clinical trial registration number: ISRCTN05212803). The study protocol was evaluated and approved by the ASRAM Institutional Review Board. An overview of the clinical study is provided in Figure 1. Briefly, 236 patients out of 823 attending the orthopaedic Outpatients Department of the ASRAM Hospital were selected, based on the signs, symptoms and radiological changes consistent with OA in the first phase of the screening procedure. A total of 75 patients suffering for more than 3 months with medial tibiofemoral OA were selected using inclusion/exclusion criteria summarized in Table 1. All patients signed the Institutional Review Board approved consent form. Patients were otherwise healthy, were aged 40 years or older, and had a diagnosis of OA, fulfilling the American College of Rheumatology classification criteria [4]. After recruitment, the patients were randomly distributed into three groups; demographic data and baseline characteristics are summarized in Table 2.

**Table 1:** Inclusion/exclusion criteria

Criteria	Details
Inclusion	Patients must understand risks and benefits of the protocol and be able to give informed consent
	Male and female patients aged 40 to 80 years
	Females of child-bearing potential must agree to use an approved form of birth control and to have a negative pregnancy test result.
	Unilateral or bilateral osteoarthritis of the knee for more than 3 months
	Visual analogue scale score during the most painful knee movement between 40 and 70 mm after 7 days of withdrawal of usual medication
	Lequesne's Functional Index score greater than 7 points after 7 days of withdrawal of usual medication
	Ability to walk
	Availability for the duration of the entire study period
	
Exclusion	History of underlying inflammatory arthropathy or severe rheumatoid arthritis
	Hyperuricaemia (>440 μmol/l) and/or past history of gout
	Recent injury in the area affected by osteoarthritis of the knee (past 4 months) and expectation of surgery in the next 4 months
	Intra-articular corticosteroid injections within the preceding 3 months
	Hypersensitivity to nonsteroidal anti-inflammatory drugs, abnormal liver or kidney function tests, history of peptic ulceration and upper gastrointestinal haemorrhage, congestive heart failure, hypertension, hyperkalaemia
	Major abnormal findings on complete blood count, history of coagulopathies, haematological or neurological disorders
	High alcohol intake (>2 standard drinks per day)
	Pregnant, breastfeeding, or planning to become pregnant during the study
	Use of concomitant prohibited medication other than ibuprofen
	Obesity (body mass index > 30 kg/m^2^)

**Table 2:** Demographic data and baseline characteristics of the patients

Characteristics	Placebo (n = 23)	100 mg/day 5-Loxin^® ^(n = 24)	250 mg/day 5-Loxin^® ^(n = 23)
Sex (male/female; n)	5/18	7/17	8/15
Age (years)	52.43 ± 9.65	52.37 ± 8.37	53.22 ± 8.73
Body weight (kg)	61.48 ± 10.69	61.08 ± 10.67	54.84 ± 10.19
Body mass index (kg/m^2^)	26.05 ± 4.29	25.91 ± 4.94	22.64 ± 4.07
Visual analog score (mm)	56.88 ± 12.04	57.05 ± 8.71	55.62 ± 9.26
Lequesne's Functional Index	12.76 ± 2.6	12.1 ± 2.76	12.04 ± 3.03
WOMAC score			
Pain subscale	38.04 ± 9.7	48.08 ± 14.05	37.17 ± 13.8
Stiffness subscale	33.15 ± 13.3	31.8 ± 17.6	27.7 ± 16.8
Function subscale	41.3 ± 9.6	41.5 ± 11.1	38.6 ± 11.1

Values are expressed as mean ± standard deviation. WOMAC, Western Ontario and McMaster Universities Osteoarthritis Index.

Before study enrollment, patients were required to be taking an NSAID at prescription strength for at least 30 days or acetaminophen 1,200 to 4,000 mg/day on a regular basis (at least 25 of the preceding 30 days) with a history of therapeutic benefit. Eligibility required patients to meet specific flare criteria upon medication washout. At screening, patients had to demonstrate a visual analog scale (VAS) score between 40 and 70 mm during the most painful knee movement, and Lequesne's Functional Index (LFI) score greater than 7 points after 7-day withdrawal of usual medication.

### Study design

A total of 75 selected patients with symptoms of moderate to mild OA were recruited into the study. Each patient was randomly assigned to a treatment group using a randomization table generated using validated computer software (RANCODE; IDV, Gauting, Germany). Treatment allocation depended only on the time sequence in which patients entered the study, thus minimizing selection bias. The clinical trial pharmacist and statistician ensured that treatment codes remained confidential. The patients were distributed into three groups: placebo (n = 25); 30% AKBA enriched *B. serrata *extract (5-Loxin^®^) low-dose group (100 mg/day), in which patients received 50 mg encapsulated 5-Loxin^® ^twice daily (n = 25); and 5-Loxin^® ^high-dose group (250 mg/day), in which patients received 125 mg encapsulated 5-Loxin^® ^twice daily (n = 25). Patients in the placebo group received two capsules of similar color, taste and appearance but filled only with rice bran.

Each patient completed a questionnaire, providing details regarding demographics, medical history and nutritional status, at the baseline evaluation and during the follow-up evaluations on days 7, 30, 60 and 90. At the baseline evaluation, and at each visit during the 90-day follow up period, all patients were assessed for pain scores and physical ability. Various parameters of serum biochemistry, haematology and urine analysis were carried out on each evaluation day. Serum samples were collected at all evaluation days for proinflammatory modulators. Knee joint synovial fluid was aseptically collected at baseline and at day 90 for evaluation of MMP-3 concentration. Safety was monitored by clinical and laboratory assessments conducted at study visits and patient-reported adverse experiences.

### Functional disability and pain score evaluation

The investigators assessed the functional disability reported by the patients at baseline and on each follow-up visit (days 7, 30, 60 and 90). Questionnaire-based assessment of pain, stiffness and physical function were done using the Western Ontario and McMaster Universities Osteoarthritis Index (WOMAC) index [20], LFI [21] and VAS [22]. The WOMAC index produces scores for three subscales: pain, stiffness and physical function. The pain, stiffness and function subscales of the WOMAC were converted to a 0 to 100 normalized units (NU) scale [23]. The pain subscale was the average of the first five questions of WOMAC and measured using the NU scale from 0 mm ('no pain') to 100 mm ('extreme pain') for each question. The stiffness subscale was the average of questions 6 and 7, measured using the NU scale from 0 mm ('no stiffness') to 100 mm ('extreme stiffness') for each question. The physical function subscale was the average of questions 8 through 24 of the WOMAC and measured by NU scale from 0 mm ('no difficulty') to 100 mm ('extreme difficulty') for each question. Analyses of these end-points were based upon the time-weighted average change from baseline over 90 days.

### Haematological and biochemical evaluations

For assessment of safety of 5-Loxin^®^, several parameters were evaluated in serum, urine and whole blood of all patients at each visit of the study duration (Table 3). Serum biochemical parameters and haematological parameters were measured using the automated analyzer HumaStar 300 (Human, Wiesbaden, Germany) and the haematological counter Humacount (Human), respectively. The urine analysis was carried out by microscopy and by using UroColor™10 Dip Sticks (Standard Diagnostics, Kyonggi-do, Korea).

**Table 3:** Parameters tested in serum biochemistry, haematology and urine analysis

Analysis	Details
Serum biochemistry	Albumin
	Alkaline phosphatase
	Total bilirubin
	Cholesterol
	Creatinine
	Creatine kinase-N-acetyl cysteine
	Glucose
	High-density lipoprotein
	Low-density lipoprotein
	Potassium
	Serum glutamic oxaloacetate transaminase
	Serum glutamate pyruvate transaminase
	Triglycerides
	Urea
Haematology	Total count and differential count
	Erythrocyte sedimentation rate
	Haemoglobin
	Platelet count
	Mean corpuscular volume
	Mean corpuscular hemoglobin
Urine analysis	Specific gravity
	pH
	Albumin
	Bile salt
	Bile pigment
	Glucose
	Red blood cell count
	Ketone bodies

### Assessment of matrix metalloproteinase-3 in synovial fluids

MMP-3 (R&D Systems, Minneapolis, USA) were quantitatively measured by ultrasensitive ELISA method. Assay procedures adhered to the protocol supplied by the manufacturers. Briefly, synovial fluid samples were incubated on capture antibody coated 96-well microplates. Specifically bound antigen was detected by appropriate biotinylated detection antibody and was probed with horseradish peroxidase enzyme. The specific immune reaction was detected by substrate solution and the colour development was read with the help of micro-plate reader (Bio-Rad, Hercules, CA, USA). A standard curve was generated by plotting the optical density at respective known concentration of MMP3. The sensitivity of MMP-3 detection ELISA kit is 9 pg/ml.

### Rescue medication

Patients were prescribed ibuprophen 400 mg tablets (maximum 400 mg thrice daily; total 1,200 mg) as rescue analgesia on days 7, 30 and 60, based on pain intensity reported to the study physician by the patient. However, the patients were instructed not to take medicine at least 3 days before each evaluation. No other OA interventions were allowed during the study period.

### Statistical analysis

We performed detailed statistical analyses using SAS software to evaluate the efficacy of two doses of 5-Loxin^® ^in comparison with the placebo group in terms of improvement in pain and physical ability scores, and to assess biomolecular markers at baseline and days 7, 30, 60 and 90 of treatment. Pair-wise changes were examined by carrying out a least significant difference test for all possible pairs. The significance of the effects of the treatment groups was compared by using one-way analysis of variance (ANOVA) followed by Tukey's multiple comparison tests. Results with *P *< 0.05 are considered statistically significant.

This is a three-arm (two doses of 5-Loxin^® ^and placebo), randomized, double-blind, placebo-controlled, single-centre trial conducted over 90 days. The trial's primary objective was to determine the effects of 5-Loxin^® ^on pain, physical function and joint stiffness. For power calculations, the estimates for variability and assumed mean changes for each treatment group were based on results from previous placebo-controlled studies of celecoxib, etoricoxib and rofecoxib conducted in patients with OA [24-27]. We believe that an intervention that gives an average improvement of mean change + 1 standard deviation, rather than mean change only, will provide results of greater significance [28]. Our trial is designed to have more than 80% power to detect a situation in which either active drug dosage yields an improvement to at least mean change + 0.9 standard deviation, under a conservative assumption, and we tested differences between groups in mean improvement using ANOVA (α = 0.05, two-sided). With 25 patients per group, we would have a 93% chance of observing at least one example of any side effect occurring in 10% or more of the patient population at a specific dosage.

## Results

### Baseline characteristics

Descriptive statistics comparing demographic variables, baseline disease characteristics and baseline outcome measures (that is, WOMAC pain, function and stiffness subscores) are provided in Table 2. Overall, the treatment groups receiving 5-Loxin^® ^low dose (100 mg/day, n = 25), 5-Loxin^® ^high dose (250 mg/day, n = 25) and placebo (n = 25), were similar with respect to sex, age, Body Mass Index and pain severity (Table 2). The patients were randomly distributed into three groups. Although there are some differences in baseline characteristics of gender, body mass index and WOMAC scores, those are statistically not significant.

### Clinical efficacy

We compared the scores between the treatment groups obtained at day 90. Both doses of 5-Loxin^® ^conferred clinically and statistically significant improvements in pain scores and physical ability scores in OA patients between baseline and day 90 (Table 4).

**Table 4:** Student's *t*-test (paired) analyses for comparison of the scores obtained from the low-dose and high-dose 5-Loxin^® ^groups at day 90

	n	Baseline		Day 90		95% CI (versus placebo)	*P*
		Mean	SD	Mean	SD		
Visual analogue scale score							
Placebo	23	56.88	12.04	41.76	15.98		<0.05
100 mg 5-Loxin^®^	24	57.05	8.71	21.37	7.13	-27.67, -13.11	<0.0001
250 mg 5-Loxin^®^	23	55.62	9.26	14.22	6.8	-34.94, -20.19	<0.0001
Lequesne's Functional Index							
Placebo	23	12.76	2.6	10.19	3.24		0.031
100 mg 5-Loxin^®^	24	12.1	2.76	7.78	4.61	-4.74, -0.07	<0.0001
250 mg 5-Loxin^®^	23	12.04	3.03	7	3.5	-5.19, -1.18	<0.0001
WOMAC pain subscale							
Placebo	23	38.04	2.03	31.74	2.58		0.1212
100 mg 5-Loxin^®^	24	42.08	2.93	19.17	3.55	-21.33, to -3.83	<0.0001
250 mg 5-Loxin^®^	23	37.17	2.88	15.22	2.50	-23.78 to -9.28	<0.0001
WOMAC stiffness subscale							
Placebo	23	33.15	2.73	24.45	2.37		0.2983
100 mg 5-Loxin^®^	24	31.77	3.61	14.06	3.71	-38.87, -6.85	<0.0001
250 mg 5-Loxin^®^	23	27.72	3.44	9.24	2.07	-43.35, -17.45	<0.0001
WOMAC function subscale							
Placebo	23	41.30	2.02	34.07	1.09		0.1048
100 mg 5-Loxin^®^	24	41.48	2.31	24.32	4.28	-18.64, -0.82	<0.0001
250 mg 5-Loxin^®^	23	38.56	2.32	17.267	1.98	-21.39, -12.23	<0.0001
MMP-3 (ng/ml)							
Placebo	15	902.1	275.6	928.5	216.02		0.4886
100 mg 5-Loxin^®^	16	893.6	270.1	637.2	224.5		<0.0001
250 mg 5-Loxin^®^	14	926.9	270.5	497.5	167.5		<0.0001

CI, confidecne interval; MMP, matrix metalloproteinase; WOMAC, Western Ontario and McMaster Universities Osteoarthritis Index.

Tukey's multiple comparison test revealed statistically significant improvements by 48.83% (*P *< 0.001), 23.79% (*P *< 0.036) and 39.61% (*P *= 0.009) in VAS, LFI and WOMAC pain scores, respectively, in the low-dose (100 mg 5-Loxin^®^) group versus the placebo group (Table 4). Improvements by 42.5% (*P *= 0.120) and 28.62% (*P *= 0.100) score in WOMAC stiffness and WOMAC functional ability, respectively were also achieved in the low-dose group (Table 4).

In comparison with the placebo group, the high-dose (250 mg 5-Loxin^®^) group also exhibited statistically significant improvements in all parameters (Table 4). The high-dose group showed improvements by 65.94% (*P *< 0.001), 31.34% (*P *< 0.017), 52.05% (*P *< 0.001), 62.22% (*P *= 0.014) and 49.34% (*P *= 0.002) in VAS, LFI, WOMAC pain, WOMAC stiffness and WOMAC functional ability scores, respectively.

**Figure 2:**
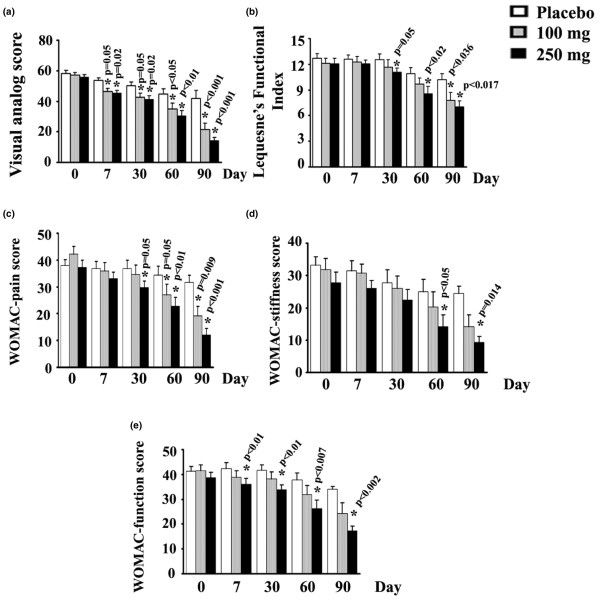
Function, pain and stiffness scores. Presented are the mean scores for **(a) **visual analog scale, **(b) **Lequesne's Functional Index, **(c) **Western Ontario and McMaster Universities Osteoarthritis Index (WOMAC)-pain, **(d) **WOMAC-stiffness, and **(e) **WOMAC-functional ability in the low-dose (100 mg/day 5-Loxin^®^) and high-dose (250 mg/day 5-Loxin^®^) groups and placebo group at different time points, as indicated. Each bar represents mean concentration ± standard deviation. In comparison with placebo, the change in scores in the treatment groups was tested for significance using Tukey's multiple comparison test; asterisk indicates statistical significance.

Student's *t*-test analyses revealed that MMP-3 concentration (*P *< 0.0001) in synovial fluids and VAS pain scores (*P *= 0.001) were significantly lower in the high-dose group than in the low-dose group. It is worth noting that both low-dose and high-dose treatment groups exhibited improvement in pain scores and physical ability scores as early as 7 days after the start of treatment, and these indices continued to improve throughout the 90 days of treatment (Figure 2). After 7 days, the low-dose and high-dose treatment groups exhibited 10.09% (*P *= 0.05) and 12.18% (*P *= 0.02) reductions in VAS, respectively, compared with the placebo group. In addition, WOMAC physical ability also improved by 14.38% (*P *< 0.01) after 7 days of treatment with high-dose 5-Loxin^® ^(Figure 2).

### Assessment of MMP-3 concentration

**Figure 3:**
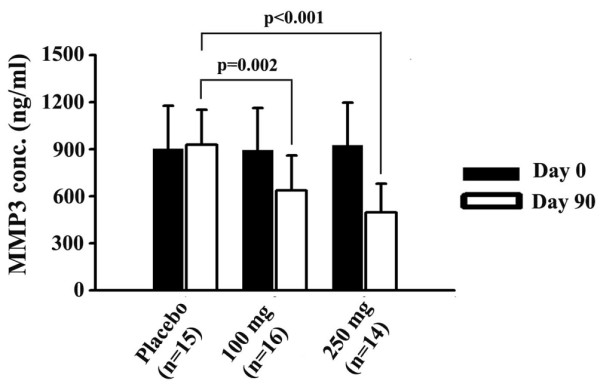
Reduction of Synovial MMP-3 levels. Presented are the matrix metalloproteinase (MMP)-3 levels in synovial fluid collected from 5-Loxin^® ^treated and placebo patients with osteoarthritis. At day 90 there was no significant change in MMP-3 concentration in the placebo group compared with baseline. In comparison with the placebo group, at the end of the study the groups receiving100 mg/day and 250 mg/day 5-Loxin^® ^showed 31.37% (*P *= 0.002) and 46.4% (*P *< 0.001) reductions in MMP-3 concentration, respectively. Change in MMP-3 concentration between the active treatment groups was not significant (*P *= 0.213). Each bar represents mean concentration of MMP-3 (ng/ml synovial fluid) ± standard deviation.

OA is a degenerative joint disorder; in molecular pathogenesis of OA, proteolytic enzymes such as MMPs are highly elevated in body fluids such as serum and synovial fluids, which cause potential damage in cartilage tissues [29]. Therefore, in order to determine whether 5-Loxin^® ^treatment can normalize the MMP level, we evaluated the concentration of MMP-3 in synovial fluids collected from the patients. Figure 3 illustrates changes in MMP-3 concentration in synovial fluid samples collected from patients of all groups included in the study. Pair-wise comparisons indicated that at the end of the study both treatment groups exhibited highly significant reductions in MMP-3 in synovial fluid. Compared with the placebo group, the low-dose (100 mg) and high-dose (250 mg) 5-Loxin^® ^groups showed 31.37% (*P *= 0.002) and 46.4% (*P *< 0.001) reductions in MMP-3 concentration, respectively. Regular *t*-tests revealed that high-dose 5-Loxin^® ^treatment significantly reduced (*P *< 0.0001) synovial MMP-3 concentration when compared with the low-dose group (Table 4). Compared with baseline, the Wilcoxon sign-rank-sum test revealed that the low-dose and high-dose groups conferred 28.69% (*P *= 0.0013) and 46.33% (*P *< 0.0001) reductions in synovial fluid MMP-3 concentration at day 90. The MMP-3 level in the placebo group remained virtually unchanged at day 90 compared with baseline.

### Biochemical evaluations

As a part of the safety evaluation, laboratory tests were performed to evaluate different biochemical parameters in serum and urine, and haematological parameters. The tested parameters in serum biochemistry, and haematological and urine analysis are summarized in Table 3. The significance of the differences between baseline and 90 days was tested by using repeated measures ANOVA. The F ratio is considered significant if *P *< 0.05. Although minor changes were observed in some of the parameters, they remained within the normal laboratory range. Statistical analyses of these parameters did not identify any statstically significant changes. Similarly, haematological and urinary parameters also exhibited no significant changes in the active treatment groups compared with placebo (data not shown). These findings further demonstrate the safety of 5-Loxin^® ^in humans.

### Adverse events

During the course of the 90-day study period, some minor adverse events were noted: diarrhoea, nausea, abdominal pain, mild fever (up to 37.5°C [99.5°F]) and general weakness. The patients who reported these minor events were distributed evenly throughout the placebo and active treatment groups. The numbers of minor adverse events reported by the patients during the study are summarized in Table 5.

**Table 5:** Incidence of adverse events

Adverse events	Placebo (n = 23)	100 mg/day 5-Loxin^® ^(n = 24)	250 mg/day 5-Loxin^® ^(n = 23)
Diarrhoea	3	2	2
Nausea	1	3	2
Vomiting	1	1	1
Abdominal pain	4	2	2
Pedal edema	0	1	0
Itching	2	1	4
General weakness	2	4	2
Constipation	0	0	1
Mild fever (up to 37.5°C)	1	2	2
Stomach burn	4	0	3
Allergy^a^	3	0	1
Headache	0	0	1
Miscellaneous^b^	9	2	6
Sum of events	30	18	27

^a^Allergy includes redness of skin and sinus allergy. ^b^Miscellaneous group includes body pain, loss of hair, chest pain and eye infection.

### Dropouts

Five patients (one from the low-dose [100 mg 5-Loxin^®^] group, and two each from placebo and high-dose [250 mg 5-Loxin^®^] group) were excluded from the study because they were suffering from a nonfatal viral infection during the course of study.

## Discussion

To the best of our knowledge, this is the first clinical study to evaluate the efficacy of 5-Loxin^® ^in OA. This study also provides important information regarding the possible molecular mechanisms of action of an anti-inflammatory compound of herbal origin in the treatment of OA. We demonstrated that 5-Loxin^® ^has potential efficacy in terms of reducing pain and improving the physical ability of OA patients. A novel aspect of the present study is its evaluation of the effect of 5-Loxin^® ^treatment on the cartilage degrading enzyme MMP-3 in synovial fluid from OA patients. In this 90-day clinical study, we also assessed the safety of 5-Loxin^® ^in OA patients.

Pain, stiffness of joints, reduced joint movement and physical disability are the major clinical manifestations of OA [1,30]. Our study demonstrates that 5-Loxin^® ^potentially improves pain, joint stiffness and physical function in OA patients (Figure 2). The statistical analyses revealed that the improvements in physical parameters and reductions in synovial MMP-3 levels were significantly decreased in the treatment groups as compared with the placebo group (Figures 2 and 3). In addition, in order to check improvements in the treatment groups, we compared the data for all parameters between the baseline and day 90. Paired *t*-test revealed that both treatment groups had highly statistically significant improvements in all parameters. Comparing the high-dose versus the low-dose groups at day 90, significant differences were observed only for VAS for pain and synovial fluid MMP3 concentration (Table 4). This finding suggests that the higher dose of 5-Loxin^® ^has better therapeutic efficacy against OA. We observed that, in comparison with baseline, there were downward trends in VAS score, LFI and WOMAC scores in the placebo group. We believe that this might be partly attributable to the placebo effect [31,32] manifesting while patients completed the questionnaires, and partly due to the consumption of ibuprophen as rescue medication by more patients in the placebo group during the study. Interestingly, at the end of the study we found that the total number of participants requesting rescue medication was 16.7% and 72.2% higher in the placebo group than in the groups receiving 100 mg and 250 mg 5-Loxin^®^, respectively.

An important observation in the present study is that 250 mg/day 5-Loxin^® ^had a significant effect in lowering VAS score by 12.18% (*P *= 0.02) and WOMAC function score by 14.38% (*P *< 0.01) in OA patients as early as 7 days after the start of treatment. These findings therefore indicate that 5-Loxin^® ^confers prompt and significant pain relief and improvement in physical ability in OA patients. Existing information reveals that glucosamine usually takes 6 weeks to achieve significant beneficial effect in terms of pain relief in OA [33]. In addition, a randomized, double-blind, placebo-controlled trial [34] showed that 4,000 mg milk protein concentrate per day (Microlactin™; Stolle Milk Biologics Inc., Cincinnati, OH, USA) yields significant improvement in WOMAC score after 2 weeks of treatment on OA patients.

In OA patients, MMPs such as MMP-3 are over-expressed and abundant in fluids of the synovial cavity, and cause degeneration of cartilage tissue [35,36]. 5-Loxin^® ^was able to reduce the elevated MMP-3 level in synovial fluid. This finding indicates that reduction in synovial fluid MMP-3 level by 5-Loxin^® ^is consistent with improvements in abnormal joint physiology in OA. Therefore, these data together demonstrate that 5-Loxin^® ^potentially has effects in terms of reducing the pain and improving physical ability and joint health; it is most likely that these improvements occur through downregulation of cartilage degrading enzymes such as MMP-3.

Earlier, in acute and subchronic toxicity studies we demonstrated that 5-Loxin^® ^(a 30% enriched product of AKBA, which is the active component of *Boswellia *extract) is safe and nontoxic in rats [15]. Additionally, 5-Loxin^® ^did not exhibit mutagenicity in the standard AMES test (INTOX; study no. 4477/05). In the present study biochemical parameters in serum, haematological parameters and urine analysis (Table 3) also did not reveal any major adverse effect of the test compound in OA patients. Taken together, these observations further demonstrate that 5-Loxin^® ^is potentially safe in the treatment of OA in humans.

## Conclusion

In summary, the present study provides the evidence in support of the potential efficacy and safety of 5-Loxin^® ^in patients with OA: 5-Loxin^® ^significantly improved joint function and exhibited better therapeutic efficacy at 250 mg/day than at 100 mg/day; it reduces pain rapidly, as early as after 1 week of treatment; it reduces levels of the cartilage degrading enzyme MMP-3 in synovial fluid; and, most importantly, 5-Loxin^® ^is safe for human consumption, even long term. This study provides important information about the efficacy and safety of 5-Loxin^® ^in the treatment of OA, which may be useful in promoting 5-Loxin^® ^as a promising alternative therapeutic strategy that may be used as a nutritional supplement against OA.

## Abbreviations

AKBA = 3-O-acetyl-11-keto-beta-boswellic acid; ANOVA = analysis of variance; ASRAM = Alluri Sitarama Raju Academy of Medical Sciences; BMI = Body Mass Index; ELISA = enzyme-linked immunosorbent assay; LFI = Lequesne's Functional Index; MMP = matrix metalloproteinase; NSAID = nonsteroidal anti-inflammatory drug; NU = normalized units; OA = osteoarthritis; VAS = visual analog scale; WOMAC = Western Ontario and McMaster Universities Osteoarthritis Index.

## Competing interests

This study is funded by Laila Impex R&D Center, India. KS, TG and KVA are employee of Laila Impex Research Centre, Vijayawada, India. ARS and SM are employee of ASRAM, Eluru, India. KVSS (SV University, Tirupati, India), DD (University of Connecticut, Storrs, CT, USA) and SPR (University of California Davis Medical Center, Davis, CA and VA Medical Center Sacramento, CA, USA) are consultants for the Laila Impex Research Center.

## Authors' contributions

KS contributed to the design of the project and data analysis, and was primarily responsible for writing the manuscript. KVA contributed to the design of the project, patient recruitment and management, and data collection. ARS and AM worked with patients to obtain informed consent, conducted clinical evaluations, took samples and evaluated therapeutic response of 5-Loxin^®^. TG contributed as the study coordinator and helped to review the manuscript. KVSS and DD helped in clinical data analysis. SPR helped in designing the study, conducting data analysis and writing the manuscript.
